# Surface plasmon resonance biosensor for exosome detection based on reformative tyramine signal amplification activated by molecular aptamer beacon

**DOI:** 10.1186/s12951-021-01210-x

**Published:** 2021-12-24

**Authors:** Wenqin Chen, Zhiyang Li, Wenqian Cheng, Tao Wu, Jia Li, Xinyu Li, Lin Liu, Huijie Bai, Shijia Ding, Xinmin Li, Xiaolin Yu

**Affiliations:** 1 Key Laboratory of Clinical Laboratory Diagnostics (Ministry of Education), College of Laboratory Medicine, Chongqing Medical University, Chongqing, 400016 China; 2 Department of Clinical Laboratory, The Affiliated Drum Tower Hospital of Nanjing University Medical School, Nanjing, 210008 China; 3 Department of Laboratory Medicine, Zigong Fourth People’s Hospital, Sichuan, 643000 China

**Keywords:** HER2-positive exosomes, Surface plasmon resonance, Tyramine signal amplification, G4-hemin, Breast cancer diagnosis

## Abstract

Human epidermal growth factor receptor 2 (HER2)-positive exosomes play an extremely important role in the diagnosis and treatment options of breast cancers. Herein, based on the reformative tyramine signal amplification (TSA) enabled by molecular aptamer beacon (MAB) conversion, a label-free surface plasmon resonance (SPR) biosensor was proposed for highly sensitive and specific detection of HER2-positive exosomes. The exosomes were captured by the HER2 aptamer region of MAB immobilized on the chip surface, which enabled the exposure of the G-quadruplex DNA (G4 DNA) that could form peroxidase-like G4-hemin. In turn, the formed G4-hemin catalyzed the deposition of plentiful tyramine-coated gold nanoparticles (AuNPs-Ty) on the exosome membrane with the help of H_2_O_2_, generating a significantly enhanced SPR signal. In the reformative TSA system, the horseradish peroxidase (HRP) as a major component was replaced with nonenzymic G4-hemin, bypassing the defects of natural enzymes. Moreover, the dual-recognition of the surface proteins and lipid membrane of the desired exosomes endowed the sensing strategy with high specificity without the interruption of free proteins. As a result, this developed SPR biosensor exhibited a wide linear range from 1.0 × 10^4^ to 1.0 × 10^7^ particles/mL. Importantly, this strategy was able to accurately distinguish HER2-positive breast cancer patients from healthy individuals, exhibiting great potential clinical application.

**Supplementary Information:**

The online version contains supplementary material available at 10.1186/s12951-021-01210-x.

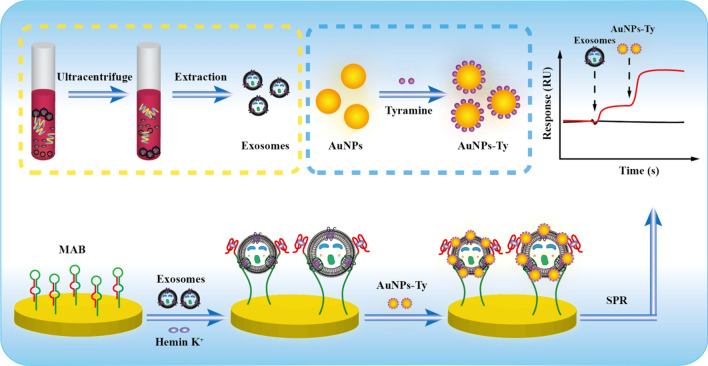

## Introduction

Breast cancer is the most common type of cancer that causes female deaths worldwide [1, 2]. In the diagnosis and treatment process of breast cancer, human epidermal growth factor receptor 2 (HER2) is an important prognostic indicator and predictor of HER2-targeted drugs [3, 4]. Currently, the routine methods for HER2 analysis mainly include immunohistochemistry and fluorescence in situ hybridization, but these methods have several shortcomings, such as the uncertainty of the results, the high false-positive rates, and the excessively harsh requirements for specimens, etc. [5]. Therefore, developing a noninvasive and reliable strategy to accurately monitor HER2 status is challenging but urgently needed.

Exosomes are 30–150 nm sized extracellular vesicles with a typical lipid bilayer membrane structure, which are secreted into the surrounding biofluids by different types of cells [6, 7]. In particular, tumor-derived exosomes not only carry cancer-specific proteins but have high abundance and stability in body fluids, thus acting as promising biomarkers for liquid biopsy [8]. Recent reports have proved that the HER2-positive exosomes are greatly increased in patients with breast cancer, and there is a good consistency of the HER2 expression level between exosomes and tumor tissues [9, 10]. Therefore, quantitating the amounts of HER2-positive exosomes has the potential to improve the diagnosis and customize treatment of breast cancer.

Apart from traditional methods including nanoparticle tracking analysis (NTA), western blot, flow cytometry, and enzyme-linked immunosorbent assay (ELISA) [11], various biosensing platforms have been developed for exosomes analysis by targeting their surface proteins using the corresponding antibodies or aptamers [12–17]. Among them, surface plasma resonance (SPR) biosensor is receiving extensive attention because it is a rapid, real-time, and label-free diagnostic device [18, 19]. However, SPR biosensor for exosomes detection faces adverse conditions: (i) the small size and low mass of exosomes cannot trigger obvious signal discrepancy, leading to the inevitable requirement of signal amplifier, and (ii) the collected exosomes are always mingled with free target proteins from serum, resulting in the generation of a false-positive signal that reduces the accuracy of results. Considering these facts, we are spurred to develop a novel SPR biosensing strategy for sensitive and specific detection of HER2-positive exosomes.

Tyramine signal amplification (TSA) is a typical enzyme-assisted amplification strategy in which tyramine can be transformed by horseradish peroxidase (HRP) into a reactive oxidized intermediate with the help of H_2_O_2_ [20]. The intermediate then covalently conjugates and rapidly deposits on near protein residues to trigger signal enhancement [21, 22]. Recently, our group proposed a cytosensor that used HRP to catalyze the deposition of tyramine-modified electroactive reporters on tumor cells, exhibiting significantly enhanced sensitivity [23]. Nevertheless, HRP has suffered from complicated purification processes and poor chemical stability due to the intrinsic characters of nature enzymes [24], which compromise the clinical applicability of TSA-based strategies. G-quadruplex-hemin (G4-hemin), an HRP-mimicking DNAzyme with excellent catalytic performance, has been widely applied in biosensing owing to its easy synthesis, high stability, and low requirement for reaction conditions [25, 26]. Therefore, G4-hemin could be a credible and promising alternative to HRP.

Herein, a label-free SPR biosensor was proposed for highly sensitive and specific detection of HER2-positive exosomes based on reformative TSA activated by target-induced molecular aptamer beacon (MAB) conversion. By combining the functions of G4 and HER2 aptamer into one molecular beacon, the engineered MABs immobilized on the sensing chip bound to HER2-positive exosomes, resulting in the structural change that accompanied the exposure of G4. Then, the formed G4-hemin catalyzed the deposition of numerous tyramine-coated gold nanoparticles (AuNPs-Ty) on the lipid membrane of the exosomes in the presence of H_2_O_2_, achieving a geometrically enhanced SPR signal. Profiting from the superior catalytic ability of G4-hemin, the improved TSA had no need for HRP. What’s more, the dual-identification of surface proteins and the membrane structure of exosomes was able to avoid the interference of the free proteins. Taken together, the developed SPR biosensing strategy enabled accurate detection of HER2-positive exosomes with high sensitivity and specificity.

## Experimental

### Reagents and materials

Sodium citrate powder, HAuCl_4_·4H_2_O and 11-mercaptoundecanoic acid (MUA) were purchased from Sinopharm Chem Co., Ltd (Shanghai, China). Hemin, tyramine, and 6-Mercapto-1-hexanol (MCH) were purchased from Sigma-Aldrich (St. Louis, USA). Hemin was dissolved in dimethyl sulfoxide as the stock solution and then diluted to the required concentration with 25 mM HEPES buffer (pH 7.4, 25 mM HEPES, 200 mM NaCl, 100 mM KCl, 1% DMSO, 0.05% Triton). Fetal Bovine Serum (FBS) and Dulbecco’s Modified Eagle Medium (DMEM) were offered by Gibco (Gaithersburg, USA). N-hydroxysuccinimide (NHS) and N-(3-(dimethylamino) propyl)-N'-ethylcarbodiimide hydrochloride (EDC) were purchased from Alfa Aesar (Massachusetts, USA). Phosphate buffer (PBS) was supplied by Thermo Fisher Scientific (Wilmington, USA). All the chemicals used were of analytical reagent grade, and all the HPLC-purified oligonucleotides listed in Additional file 1: Table S1 were prepared by Sangon Biotech. Co., Ltd (Shanghai, China). Tris–EDTA (TE) buffer (10 mM Tris–HCl, 1 mM EDTA, pH 8.0) was utilized for the dissolution of oligonucleotides. Aqueous solutions prepared by Millipore Milli-Q gradient ultrapure water system were used (Millipore Co., MA, USA). Clinical samples were collected from the First Affiliated Hospital of Chongqing Medical University.

### Instruments

All measurements were carried out on the SPR biochemical analyzer independently developed and built by our group. The sensing principle of this technique is to measure the change in refractive index around the surface of the metallic sensor chip due to alterations in mass caused by analyte-receptor noncovalent interactions [27, 28]. The SPR analyzer with a signal acquisition frequency of 5 s is mainly composed of a light source, a sensing chip, a flow cell, and a CCD detector. The sensorgrams are analyzed by the lab-developed program written in LabVIEW, exhibiting a time course of resonance units (RU). Transmission electron microscopic (TEM) image was performed with an H-7500 transmission electron microscope (Hitachi High-Technologies Co., Japan). Nanoparticles tracking analysis (NTA) was supported by ZetaView (Particle Metrix, Germany). UV–Visible absorption spectra were carried out at a UV-2550 spectrophotometer (Shimadzu, Japan).

### Extraction of exosomes

All cell lines, including HER2-positive SK-BR3 and HER2-negative HeLa, LNCaP, HepG2, and MCF-7 [29–32], were provided by the American Type Culture Collection (ATCC) (Rockville, USA). DMEM (added with 10% FBS, 1% streptomycin and penicillin) was used for all kinds of cell culture in a sterile incubator containing 5% CO_2_, at 37 ºC. Cell passaging was processed when growth density reached 80%. Ultimately, the cells were starved with serum-free medium for 48 h before the exosomes were extracted.

The exosomes were extracted from the cell culture medium according to the published works [33, 34]. First of all, the cell culture supernatant was collected and centrifuged (10,000×*g*, 30 min). This pretreatment process could separate the exosomes from larger vesicles and cell debris for preliminary purification. After centrifugation, the supernatant was collected and centrifuged again (100,000×*g*, 70 min) to extract the exosomes secreted by tumor cells. After that, the obtained exosomes were washed with 1 × PBS to remove protein impurities, and centrifuged again for further purification (100,000×*g*, 70 min). After that, the obtained exosomes were resuspended in 100 µL of PBS and stored at −80 ºC for later use. The extraction procedures of exosomes derived from serum are as follows. To begin with, 1 mL of PBS was added to 1 mL of serum and then centrifuged (12,000×*g*, 45 min). Afterward, the obtained supernatant was centrifuged again (110,000×*g*, 2 h). After that, the supernatant was discarded, and the exosomes precipitated at the bottom were suspended in 8 mL of PBS solution and passed through a 0.22 µm filter membrane to remove impurities with smaller particle diameters. Thereafter, the filtrate was centrifuged again (110,000×*g*, 70 min) for further purification. Finally, the pure exosomes obtained after centrifugation were resuspended in 100 µL of PBS and stored at −80 ºC for later use. TEM and NTA were performed to characterize the obtained cancer cell-derived exosomes.

### Preparation of AuNPs-Ty complex

Firstly, AuNPs with a particle size of about 14 nm were synthesized according to the published works with slight modification [35–39]. Briefly, 88.2 mL of HAuCl_4_ (1 mM) was boiled and then added rapidly to 10.1 mL of sodium citrate (34 mM). After the color changed to burgundy, the heating was stopped, and the mixture was cooled to room temperature with continued stirring. Then the prepared AuNPs and tyramine were combined by MUA that has sulfhydryl and carboxyl groups at each end. In detail, the sulfhydryl groups at one end of MUA were connected to AuNPs through Au–S bonds, and the other end was connected to tyramine through carboxyl groups, which indirectly connected AuNPs and tyramine into AuNPs-Ty complex. To begin with, MUA (25 μL, 100 μM) was added to the AuNPs (5 mL) solution, gently shaking for 12 h, and then the solution was centrifuged twice (2000×*g*, 10 min) to get AuNPs-MUA solution. Thereafter, NHS (10 μL, 1 mg/mL), EDC (10 μL, 2 mg/mL) and tyramine (10 μL, 100 μM) were added to the mixed solution, gently shaking for 12 h to get AuNPs-Ty complex, which was stored at 4 ℃ for later use.

### Exosomes analysis by the fabricated SPR biosensor

Firstly, the gold chip and the flow cell were immersed in 75% ethanol, followed by sonication for 20 min at room temperature. Then the chip was rinsed with deionized water and blew dry with nitrogen. Next, the piranha solution 1 mL (H_2_SO_4_: 700 μL, H_2_O_2_: 300 μL) was freshly prepared, after cooling to room temperature, we covered it on the chip surface for 10 min to remove the remaining impurities. Again, the chip was rinsed thoroughly with deionized water and blew dry with nitrogen. Next, the capture probes of MAB were diluted to a concentration of 1.5 μM by the fixative solution (KH_2_PO_4_, 1 M), and then modified on the surface of the chip overnight at 4 ℃. After the incubation, 1 mM MCH was used to block the excess binding sites for 30 min. After washing and drying, the treated chip, the prism cleaned with 75% ethanol, and the flow cell were installed on the SPR instrument for subsequent testing. Thereafter, the flow pipeline was rinsed with cell grade 1 × PBS for 15 min to output the baseline signal (flow rate: 50 μL/min). Then, different concentrations of exosomes derived from tumor cells or clinical samples were loaded (loading volume: 150 μL, flow rate: 7 μL/min, time: 30 min). Subsequently, after the signal was stabilized, the AuNPs-Ty composite was loaded (loading volume: 150 μL, flow rate: 7 μL/min, time: 30 min). After detection, the surface of the sensing chip could be regenerated with 50 mM NaOH (loading volume: 150 μL, flow rate: 20 μL/min, time: 2 min).

## Results and discussion

### Illustration of the SPR biosensing strategy

**Scheme 1:**
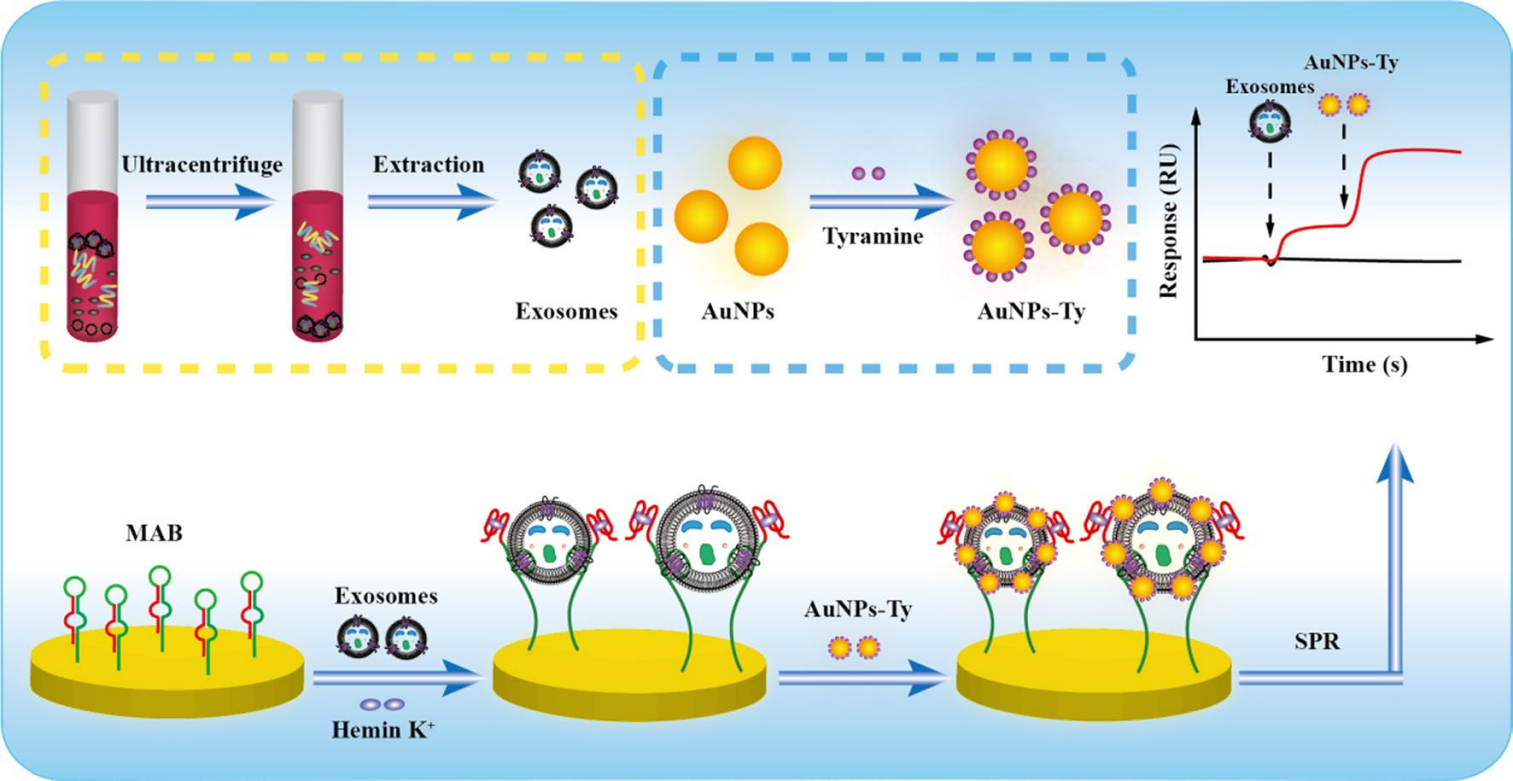
Schematic illustration for the detection of HER2-positive exosomes based on the improved TSA enabled by target-induced MAB conversion

Scheme 1 clearly showed the detection procedures of HER2-positive exosomes using the reformative TSA activated by target-induced MAB transition. Specifically, exosomes were extracted from the cell culture fluid or clinical samples by ultracentrifugation, and the SPR signal amplifier of AuNPs-Ty was prepared by conjugating the tyramine molecules to AuNPs. Meanwhile, the ingeniously designed MAB containing HER2 aptamer (green domain) and G4 (red domain) sequences was modified on the surface of the gold chip by Au–S bond. In the presence of HER2-positive exosomes, the MAB captured the exosomes by the interaction of aptamer and HER2 protein, which led to the exposure of G4. The unlocked G4 formed catalytically active G4-hemin with the assistance of hemin and K^+^. Subsequently, the activated G4-hemin prompted a large amount of AuNPs-Ty to in situ deposit on the surface of the exosomal membrane through the TSA process, resulting in the production of a greatly enhanced SPR signal. As a result, the developed SPR strategy achieved label-free, real-time, and highly specific detection of low-abundance HER2-positive exosomes.

### Characterization of the extracted exosomes, G4-hemin, and AuNPs-Ty

**Fig. 1:**
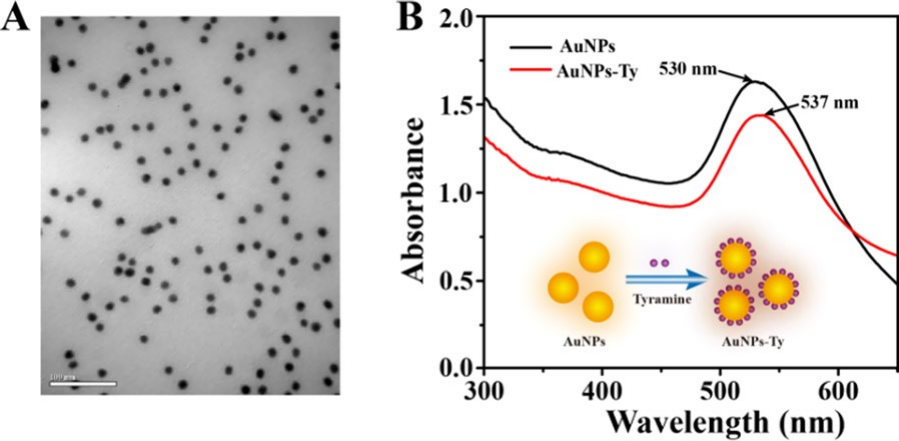
Characterization of the signal amplifier of AuNPs-Ty. **A** TEM image of AuNPs. Scale bars = 100 nm, **B** UV–vis absorption spectra of AuNPs (black line) and AuNPs-Ty (red line)

First, the morphology and concentration of exosomes derived from SK-BR3 cells were characterized, and the results were shown in Additional file 1: Fig. S1. The TEM image disclosed that the exosomes were round vesicles with a clear double-layer membrane structure (Additional file 1: Fig. S1A). The NTA indicated that the original concentration of the exosomes was 2.75 × 10^9^ particles/mL with a size distribution ranging from 30 to 200 nm (Additional file 1: Fig. S1B). The above results confirmed the successful extraction of exosomes. Second, the combination of G4 and hemin was verified by UV–vis absorption spectroscopy. As we can learn from Additional file 1: Fig. S2, 260 nm and 394 nm were the characteristic absorption peaks of G4 and hemin, respectively. As expected, in the absorption spectrum of G4-hemin, the absorption peaks of the two substances with a slight right shift were observed at the same time, indicating the successful formation of G4-hemin. Third, the characterization of AuNPs-Ty composite was performed. The TEM image showed the prepared AuNPs were uniform in volume and evenly distributed (Fig. 1A), and the UV–vis absorption spectra displayed that the typic absorption peaks of AuNPs and AuNPs-Ty were seen at 530 nm and 537 nm, respectively (Fig. 1B). These results were entirely consistent with the previous report [24], indicating the successful synthesis of AuNPs-Ty.

### Feasibility of the developed biosensor

**Fig. 2:**
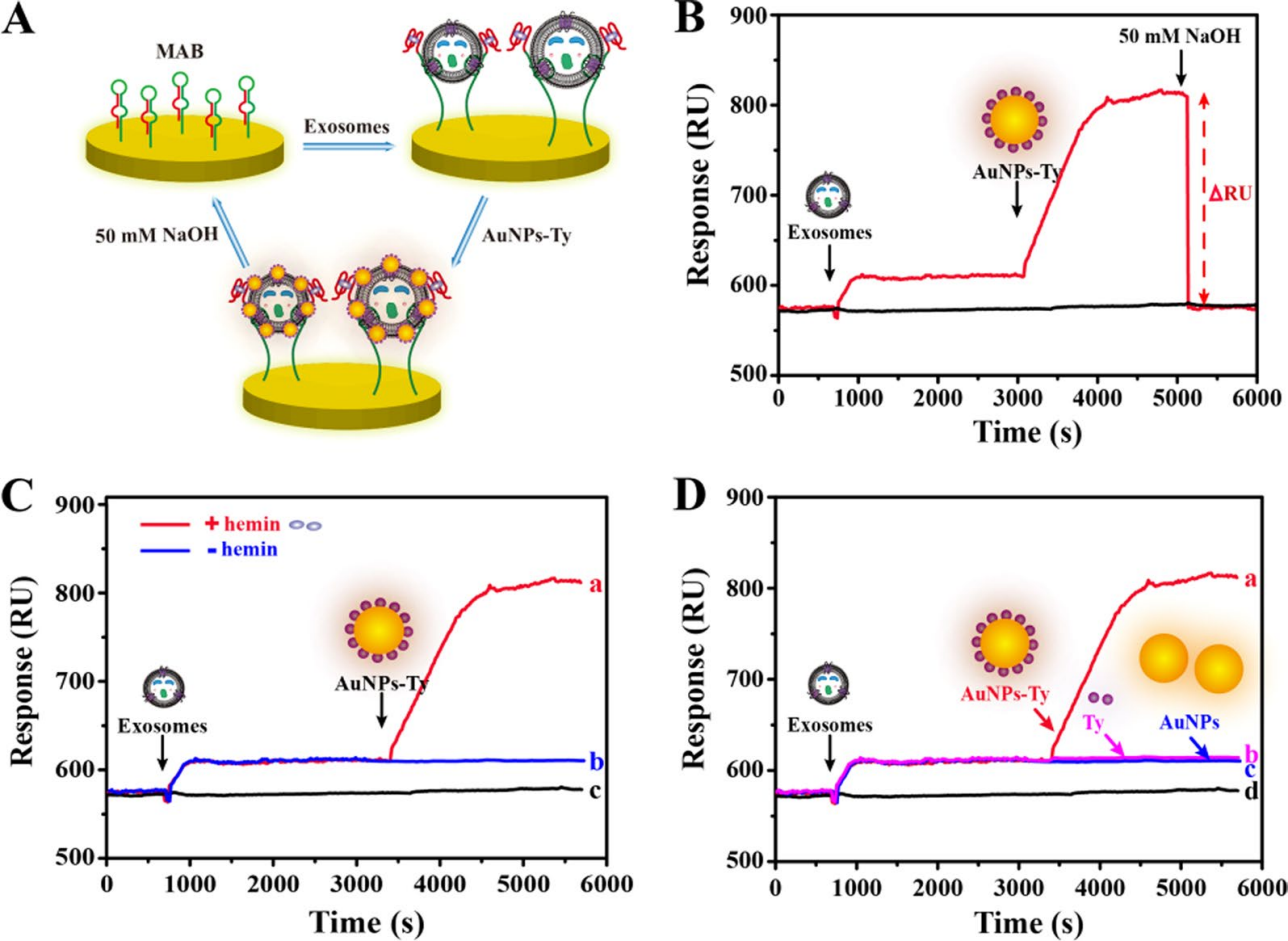
Evaluation of the feasibility of the biosensing strategy. **A** Schematic diagram of the detection process. **B** Typical SPR sensorgrams of the biosensor (red line) and blank control without loading (black line). **C** SPR sensorgrams of the different conditions: (a) the intact sensing system, (b) the sensing system without hemin, and (c) blank control. **D** SPR sensorgrams of the different materials: (a) AuNPs-Ty, (b) Ty, (c) AuNPs, and (d) blank control. The concentration of HER2-positive exosomes derived from SK-BR3 cells is 1 × 10^7^ particles**/**mL. All data expressed as mean ± standard variation (n = 3)

To demonstrate the feasibility of this proposed biosensor, the whole detection process was firstly analyzed. The detection steps and the corresponding sensorgrams were depicted in Fig. 2A and B, respectively. In the first step, when the HER2-positive exosomes were loaded, the SPR signal rose by about 40 RU, indicating that the exosomes were connected to the surface of the chip owing to the effective binding of the aptamer of the MAB and HER2 proteins. In the second step, once the AuNPs-Ty composite was loaded, the SPR signal increased by about 200 RU, which was attributed to the deposition of plentiful AuNPs-Ty on the surface of the exosomal membrane caused by the reformative TSA. After detection, the chip surface was regenerated with 50 mM NaOH for next measurement. Subsequently, to verify that the deposition of AuNPs-Ty on the exosome surface was caused by the catalytic activity of G4-hemin rather than the nonspecific absorption, the relevant experiments were conducted. As shown in Fig. 2C, in the absence of hemin, the signal of the AuNPs-Ty was not been observed due to the fact that the exposed G4 had not peroxidase-like activity. In contrast, the AuNPs-Ty generated a distinct SPR signal, demonstrating that the signal indeed originated from the reformative TSA. In addition, compared with Ty and AuNPs that produced negligible SPR signal, the signal of AuNPs-Ty increased nearly tenfold (Fig. 2D). These results adequately demonstrated the feasibility of the proposed method for the detection of HER2-positive exosomes.

### Optimization of experimental parameters

Several important reaction parameters of this biosensor were optimized to obtain good analytical performance in the presence of 1.0 × 10^7^ particles/mL HER2-positive exosomes. Owing to the effect of the density of capture probe immobilized on the sensing chip on the sensitivity of the biosensor, the concentration of the MAB (C_MAB_) was firstly optimized. As shown in Additional file 1: Fig. S3A, the SPR signal gradually improved with the increase of the C_MAB_ ranging from 0.5 to 1.5 μM. Once the concentration was above 1.5 μM, the signal decreased rapidly, because the crowded MAB could not bind to the exosomes effectively. Therefore, 1.5 μM was chosen as the optimal C_MAB_. When the concentration of hemin (C_hemin_) was 1.5 μM, being the same as the C_MAB_, the highest signal was obtained. Hence, the optimal C_hemin_ is set to 1.5 μM (Additional file 1: Fig. S3B).

Furthermore, to avoid the nonspecific deposition of redundant signal amplifier on the chip surface which increased the background signal, the befitting concentration of the AuNPs-Ty (C_AuNPs-Ty_) was evaluated. As shown in Additional file 1: Fig. S3C, when the C_AuNPs-Ty_ rose from 50 to 200 mM, the SPR signal rose synchronously and ended by a downward trend. Therefore, we chose 200 mM as the optimal C_AuNPs-Ty_. Subsequently, we also optimized the reaction time between exosomes and MAB (Time_1_) and the incubation time of the improved TSA (Time_2_), respectively (Additional file 1: Fig. S3D and E). When Time_1_ was set as 30 min, the signal reached the peak and remained steady as time prolonged, indicating the completion of the reaction. Similarly, in the optimization experiment of Time_2_, the reaction was completed after 30 min. Therefore, we set both reaction times as 30 min.

### Sensitivity of the SPR biosensor

**Fig. 3:**
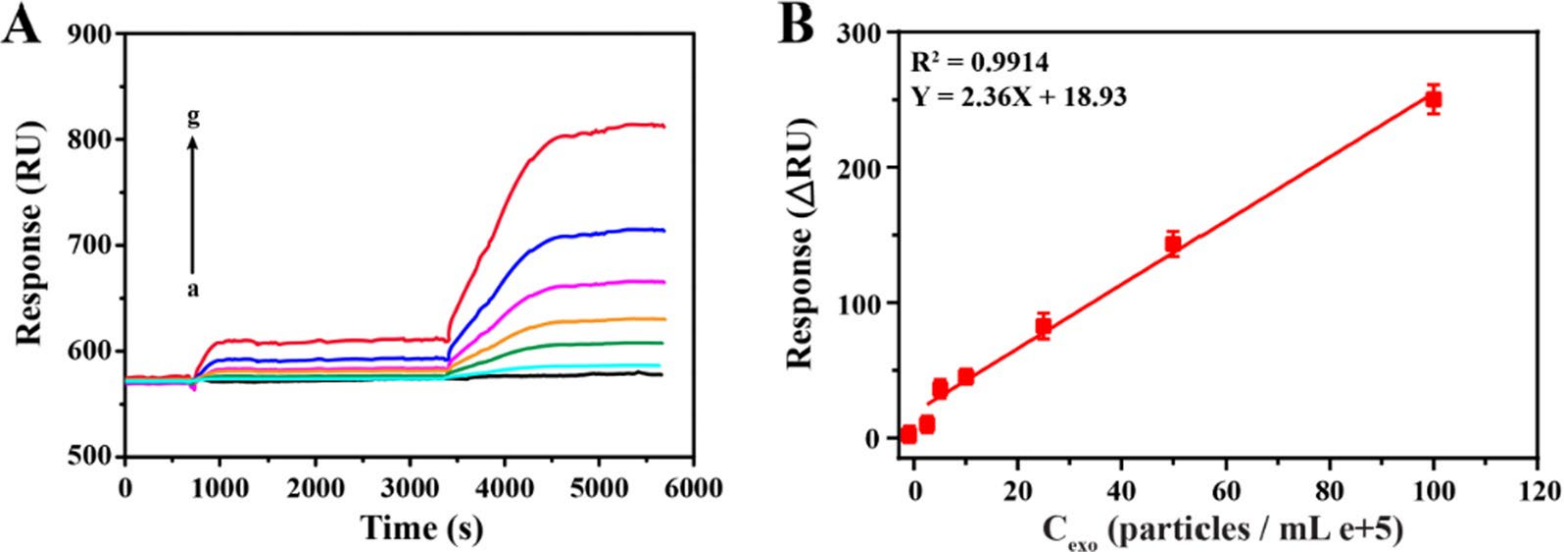
Evaluation of the sensitivity of the SPR biosensor. **A** SPR sensorgrams and (**B**) the calibration line for exosomes at different concentrations of 0.1, 2.5, 5, 10, 25, 50, 100 particles/mL e + 5 (from a to g). All data expressed as mean ± standard variation (n = 3)

Under the optimal reaction conditions, we further evaluated the sensitivity of this sensing method by detecting a series of different concentrations of HER2-positive exosomes. As shown in Fig. 3A, when the concentration of exosomes increased from 0.1 to 100 × 10^5^ particles/mL, the SPR signal also increased simultaneously, showing a good correlation. As presented in, the regression equation between the concentration of exosomes (X) and the corresponding SPR signals (Y) was Y = 2.36 X + 18.93 with a correlation coefficient of 0.9914 (Fig. 3B). The lowest detectable concentration of the sensing strategy was 1.0 × 10^4^ particles/mL, which was comparable to other biosensing strategies for exosomes detection, and the detailed comparison was shown in Additional file 1: Table S2. The excellent analytical performance of this method is mainly due to these aspects. First, the high affinity of the aptamer to HER2 proteins allowed the G4 sequence to be fully exposed to form the G4-hemin. Second, the formed G4-hemin exerted superior peroxidase-like activity itself to catalytic the in situ deposition of numerous AuNPs-Ty on the exosomes, resulting in the high SPR signal.

### Specificity of the SPR biosensing strategy

**Fig. 4:**
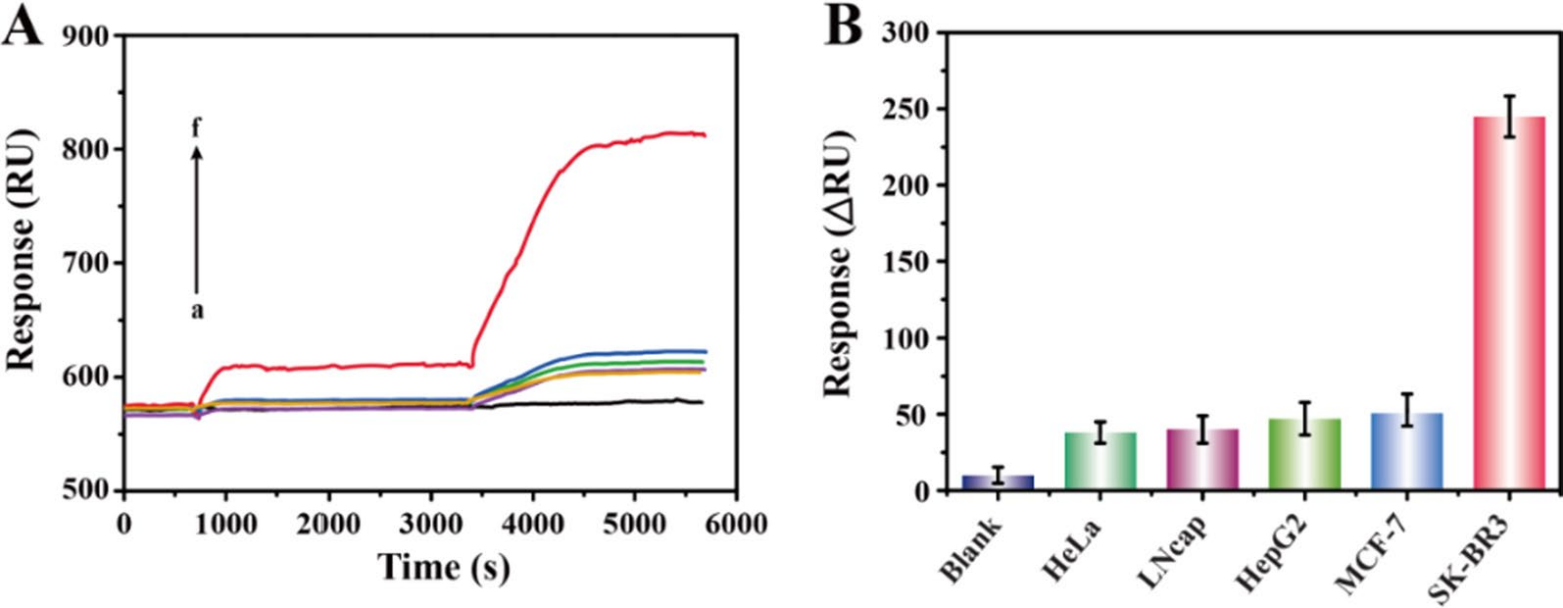
Evaluation of the specificity of the sensing strategy. **A** SPR detection sensorgrams and (**B**) the response signals of (a) blank control and exosomes (1.0 × 10^7^ particles**/**mL) derived from (b) HeLa, (c) LNcap, (d) HepG2, (e) MCF-7, and (f) SK-BR3 cell lines, respectively. All data expressed as mean ± standard variation (n = 3)

The good specificity is one of the indispensable characteristics of an excellent biosensor, which directly affects the clinical application prospects of this method. Accordingly, we conducted follow-up researches to explore the specificity of this sensing strategy by choosing another four kinds of exosomes derived from different HER2-negative cancer cells (HeLa, LNcap, HepG2, and MCF-7) as interferents. As shown in Fig. 4, compared with these interferents, exosomes from HER2-positive SK-BR3 cells produced a significantly improved SPR signal. In general, the SPR signal of HER2-positive exosomes was 4.9 to 6.4 times to those of interfering substances at the same concentration (1.0 × 10^7^ particles**/**mL). Besides, by detecting exosomes using the developed strategy, the cancer cells with high HER2 expression could be discriminated accurately. These results showed the excellent specificity of this method, which was mainly attributed to the dual-identification of HER2 proteins and the lipid membrane of exosomes.

### Detection of exosomes in clinical samples

**Fig. 5:**
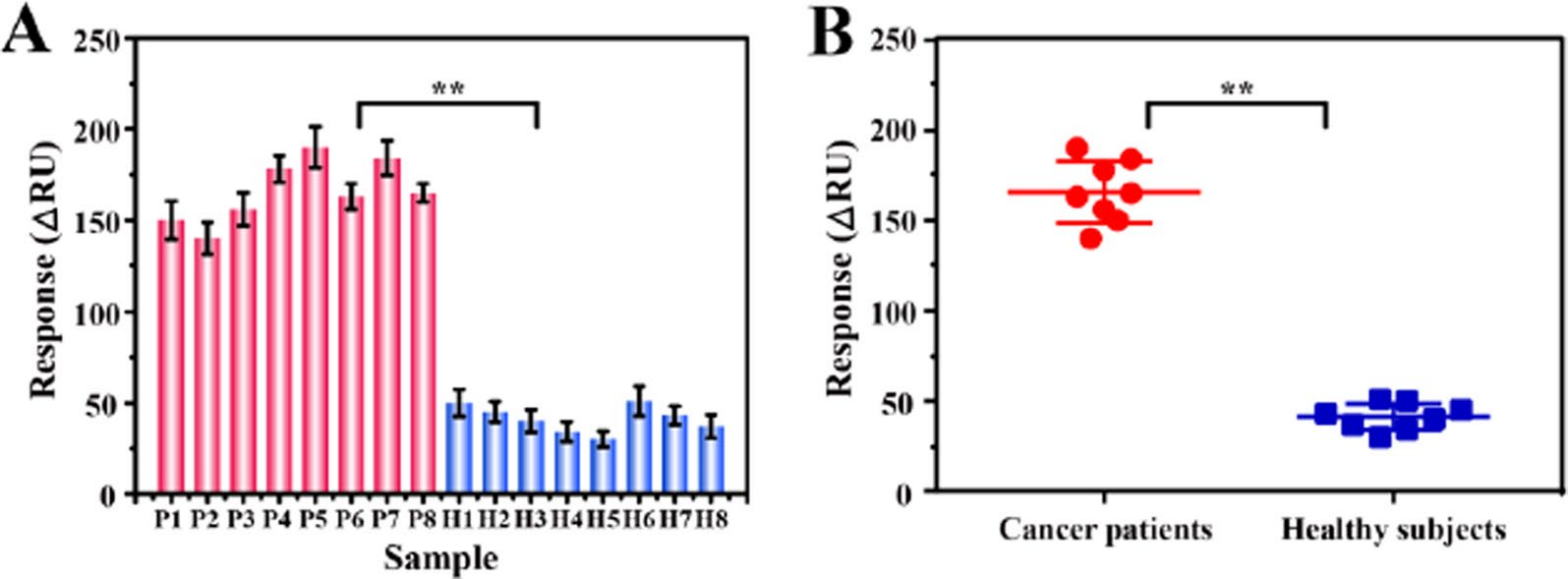
Investigation of the clinical applicability of this biosensor. **A** Analysis of exosomes from clinical samples (breast cancer patients: P1–P8 and healthy control subjects: H1–H8) by the developed strategy and (**B**) the corresponding scatter plot. Significance was determined by Student unpaired t-test, ** *p* < 0.05

To evaluate the applicability of this method for the diagnosis of breast cancer, exosomes derived from real clinical samples were analyzed. We collected 16 serum samples from the First Affiliated Hospital of Chongqing Medical University. Among them, half of the samples were from HER2-positive breast cancer patients (P1–P8), and the rest were from healthy groups (H1–H8), which served as testing control. As shown in Fig. 5A, almost all SPR signals obtained by detecting exosomes from the serum of HER2-positive breast cancer patients were greater than 150 RU (except for P2), however, under the same circumstances, the signals obtained by detecting exosomes from healthy samples were all lower than 50 RU. The different signal levels of the positive samples were due to the fact that patients with different stages could secrete various concentrations of HER2-positive exosomes. In addition, the calculation of the significance probability P-value also illustrated the significant difference between the two groups of data (Fig. 5B), implying the outstanding distinction ability of this method between HER2-positive breast cancer patients and healthy groups. These results shed light on the potential clinical application prospect of this sensing method.

## Conclusions

In conclusion, a label-free SPR biosensor has been developed for highly sensitive and specific detection of HER2-positive exosomes based on the reformative TSA activated by target-induced MAB conversion. Different from the traditional TSA, the reformative TSA replacing HRP with G4-hemin fully avoids the intrinsic disadvantages of natural enzymes, thus possessing a high potential for clinical transformation. And the biosensing strategy is able to simultaneously recognize HER2 proteins and the lipid membrane of the exosomes, which effectively eliminates the interference of free proteins in purified exosomes. Benefiting from the integrating of the MAB and the reformative TSA, large quantities of AuNPs-Ty are in-situ deposited on the HER2-positive exosome membrane, endowing the developed SPR biosensor with high sensitivity and specificity. Moreover, by simply changing the aptamer types, this sensing strategy can be easily extended for accurate detection of other exosome subtypes. More importantly, this method has capable of discerning patients with HER2-positive breast cancer from healthy individuals. Overall, this work offers a new SPR platform for exosome-based liquid biopsy in the diagnosis of breast cancer. Despite these results, a limitation of this method is that the experimental steps need to be further simplified to improve the flexibility in clinical applications.

## Supplementary Information

**Additional file 1: Fig. S1**. Characterization of the exosomes. (A) TEM image and (B) NTA of the collected exosomes derived from SK-BR3 cells. **Fig. S2.** Characterization of G4-hemin. UV–vis absorption spectra of free hemin (black line), G4 (red line), and G4-hemin (blue line). **Fig. S3.** Optimizations of the concentrations of (A) the MAB, (B) hemin, (C) AuNPs-Ty, the reaction time of (D) MAB and exosomes, and (E) the incubation time of the developed TSA. The concentration of exosomes is 1.0 × 10^7^ particles/mL. **Table S1.** Sequences of oligonucleotides employed in this work. **Table S2.** Comparison of biosensing strategies for the detection of exosomes.

## Data Availability

All data generated and analyzed during this study are included in this article and additional file. The additional file is available. Characterization of the exosomes, characterization of G4-hemin, and Optimizations of reaction conditions (Additional file 1: Figs. S1–S3). Sequences of oligonucleotides employed in this work (Additional file 1: Table S1). Comparison of biosensing strategies for the detection of exosomes (Additional file 1: Table S2).

## Abbreviations

TSA: Tyramine signal amplification
MAB: Molecular aptamer beacon
SPR: Surface plasmon resonance
AuNPs-Ty: Tyramine-coated AuNPs
HRP: Horseradish peroxidase
HER2: Human epidermal growth factor receptor 2
AuNPs: Gold nanoparticles
G4-hemin: G-quadruplex-hemin
MCH: 6-Mercapto-1-hexanol
FBS: Fetal Bovine Serum
DMEM: Dulbecco’s Modified Eagle Medium
NHS: N-hydroxysuccinimide
EDC: N-(3-(dimethylamino) propyl)-N'-ethylcarbodiimide hydrochloride
PBS: Phosphate buffer
RU: Resonance units
TEM: Transmission electron microscopic
NTA: Nanoparticles tracking analysis
