# Supplementary material for: Surface plasmon resonance biosensor for exosome detection based on reformative tyramine signal amplification activated by molecular aptamer beacon
Source: J Nanobiotechnology. 2021 Dec 24;19:450. doi: 10.1186/s12951-021-01210-x (PMC8709980; doi:10.1186/s12951-021-01210-x)
Supplement: Supplementary file 1 — Additional file 1: Fig. S1. Characterization of the exosomes. (A) TEM image and (B) NTA of the collected exosomes derived from SK-BR3 cells. Fig. S2. Characterization of G4-hemin. UV–vis absorption spectra of free hemin (black line), G4 (red line), and G4-hemin (blue line). Fig. S3. Optimizations of the concentrations of (A) the MAB, (B) hemin, (C) AuNPs-Ty, the reaction time of (D) MAB and exosomes, and (E) the incubation time of the developed TSA. The concentration of exosomes is 1.0 × 10^7^ particles/mL. Table S1. Sequences of oligonucleotides employed in this work. Table S2. Comparison of biosensing strategies for the detection of exosomes. [file 12951_2021_1210_MOESM1_ESM.docx]

**Additional file 1**

**Surface plasmon resonance biosensor for exosome detection based on reformative tyramine signal amplification activated by molecular aptamer beacon**

*Wenqin Chen ^a, b, 1^, Zhiyang Li ^b, 1^, Wenqian Cheng ^a^, Tao Wu ^c^, Jia Li ^a^, Xinyu Li ^a^, Lin Liu ^c^, Huijie Bai ^a^,* *Shijia Ding ^a^, Xinmin Li ^a,^ *,* *Xiaolin Yu ^c,^* ***

*^a^ Key Laboratory of Clinical Laboratory Diagnostics (Ministry of Education), College of Laboratory Medicine, Chongqing Medical University, Chongqing 400016, China*

*^b^* *Department of Clinical Laboratory, the Affiliated Drum Tower Hospital of Nanjing*

*University Medical School, Nanjing 210008, China*

*^c^ Department of Laboratory Medicine, Zigong Fourth People’s Hospital, Sichuan 643000, China*

*** Corresponding author**

E-mail: E-mail: yuxiaolincq@hotmail.com (X. Yu); xinmnli@163.com (X. Li).

**Author Contributions**^1^ W. Chen and Z. Li contributed equally to this work.

**
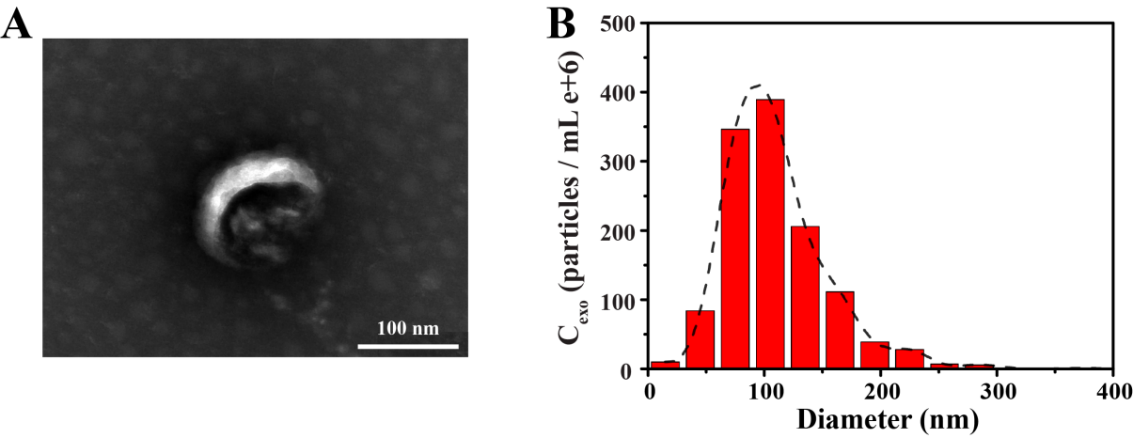
**

**Fig. S1** Characterization of the exosomes. (A) TEM image and (B) NTA of the collected exosomes derived from SK-BR3 cells.


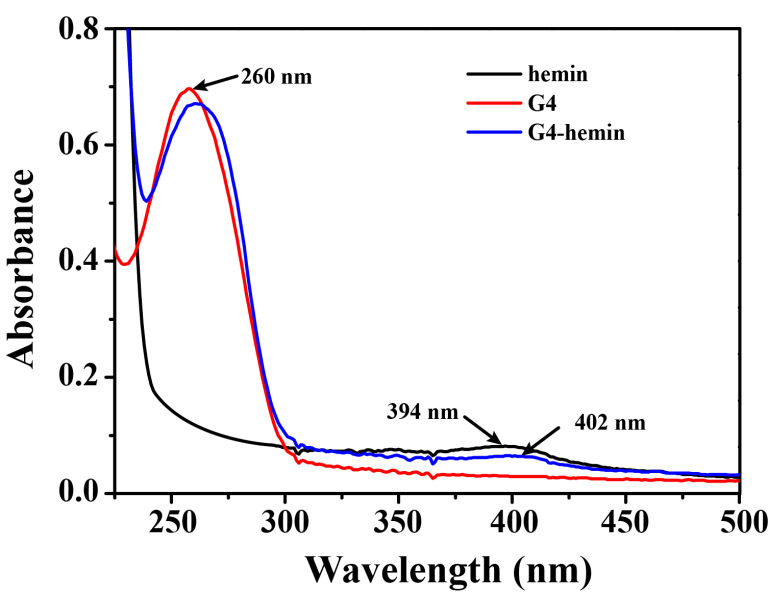


**Fig. S2** Characterization of G4-hemin. UV–vis absorption spectra of free hemin (black line), G4 (red line), and G4-hemin (blue line).

**
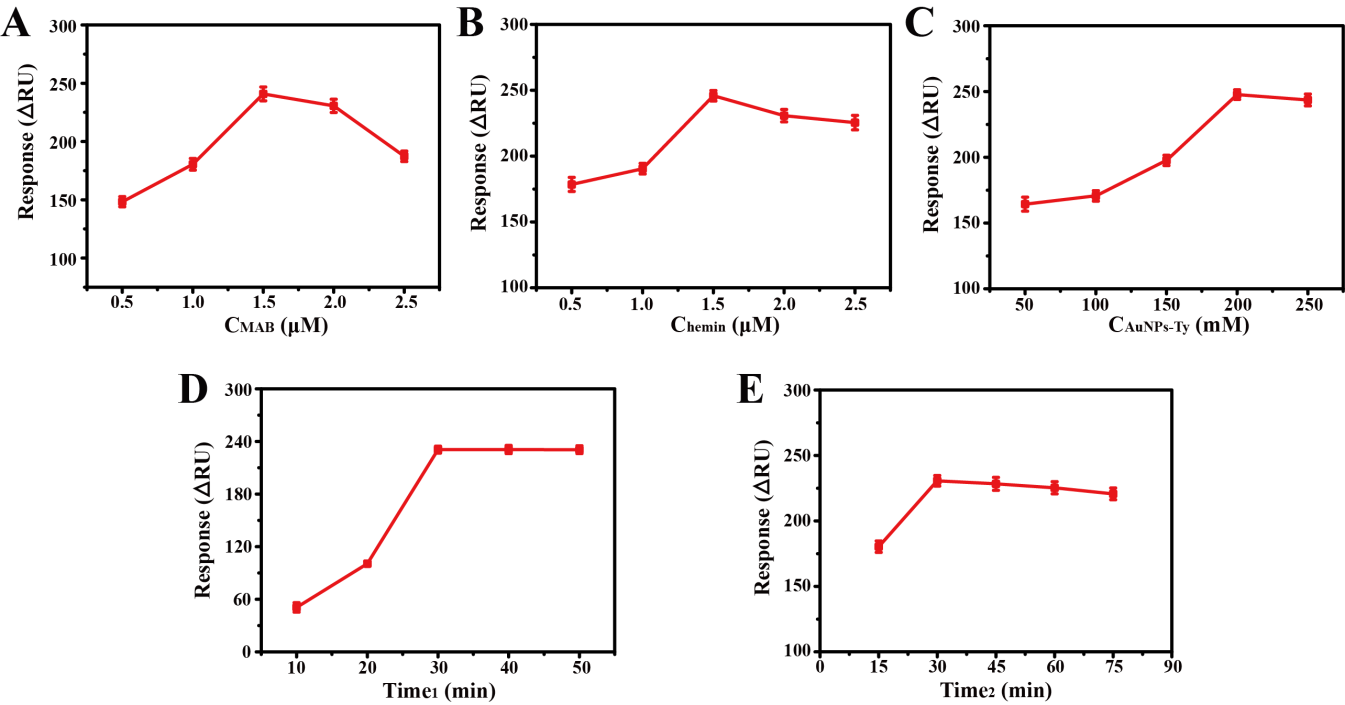
**

**Fig. S3** Optimizations of the concentrations of (A) the MAB, (B) hemin, (C) AuNPs-Ty, the reaction time of (D) MAB and exosomes, and (E) the incubation time of the developed TSA. The concentration of exosomes is 1.0 × 10^7^ particles/mL.

**Table S1.** Sequences of oligonucleotides employed in this work.

| Oligonucleotides | Sequences (5’-3’) |
| --- | --- |
| Hairpin probe | GTACTCGGGTGGGTGGGTGGGTCCACGCAGGGCCGTCGAACACGAGCATGGTGCGTGGACCTAGGATGACCTGAGTACTGTCCT_15_- C_6_HS-SH |

**Table S2.** Comparison of biosensing strategies for the detection of exosomes.

| **Detection platform** | **Amplification strategy** | **Linear range (particle/mL)** | **LOD (particle/mL)** | **Ref.** |
| --- | --- | --- | --- | --- |
| Colorimetry | Enzyme-induced etching of Au NBP@MnO_2_ NSs | 8.50 × 10^5^- 8.50 × 10^7^ | 1.35 × 10^5^ | [1] |
| Fluorescence | Branched RCA | 1.00 × 10^5^- 1.00 ×10^9^ | 4.27 × 10^4^ | [2] |
| Electrochemistry | NTH-coupled enzymatic signal amplification | 2.16 × 10^4^- 7.50 × 10^7^ | 1.66 × 10^4^ | [3] |
| Fluorescence | Fe_3_O_4_@SiO_2_@TiO_2_ particles | 5.00 × 10^5^- 1.00 × 10^7^ | 5.00 × 10^5^ | [4] |
| Colorimetry | DNAzyme | 8.30 × 10^5^- 5.30 × 10^7^ | 3.94 × 10^5^ | [5] |
| SERS | Gold Nanorods | 1.00 ×10^6^-1.00 ×10^8^ | 2.00 × 10^6^ | [6] |
| SPR | MAB and the reformative TSA | 1.00 × 10^4^ -1.00 × 10^7^ | 1.00 × 10^4^ | This  work |
| Au NBP@MnO_2_ NSs: gold nanobipyramid@MnO_2_ nanosheet nanostructures; RCA: rolling circle amplification; NTH: DNA nanotetrahedron; SERS: Surface-Enhanced Raman Scattering; SPR: surface plasmon resonance; MAB: molecular aptamer beacon; TSA: tyramine signal amplification. | | | | |

**References**

[1] Huang R, He L, Li S, Liu H, Jin L, Chen Z, Zhao Y, Li Z, Deng Y, He N. A simple fluorescence aptasensor for gastric cancer exosome detection based on branched rolling circle amplification. Nanoscale. 2020; 12(4):2445-2451.

[2] Jiang J, Yu Y, Zhang H, Cai C. Electrochemical aptasensor for exosomal proteins profiling based on DNA nanotetrahedron coupled with enzymatic signal amplification. Anal. Chim. Acta. 2020; 1130:1-9.

[3] Li Q, Wang Y, Ling L, Qiao L, Chen H, Ding C, Yu S. Rapid and specific detection nanoplatform of serum exosomes for prostate cancer diagnosis. Microchim. Acta. 2021; 188 (8): 283.

[4] Zhang Y, Jiao J, Wei Y, Wang D, Yang C, Xu Z. Plasmonic Colorimetric Biosensor for Sensitive Exosome Detection via Enzyme-Induced Etching of Gold Nanobipyramid@MnO_2_ Nanosheet Nanostructures. Anal. Chem. 2020; 92 (22):15244-15252.

[5] Zhou Y, Xu H, Wang H, Ye B. Detection of breast cancer-derived exosomes using the horseradish peroxidase-mimicking DNAzyme as an aptasensor. Analyst 2019; 145 (1): 107-114.

[6] Kwizera EA, O’Connor R, Vinduska V, Williams M, Butch ER, Snyder SE, Chen X, Huang XH, Theranostics. 2018; 8 (10): 2722-2738.
